# Progress in Diabetes

**Published:** 1901-01-26

**Authors:** 


### PROGRESS IN DIABETES.

**Pathology.**—Pavy[1] has proposed the classification of ordinary forms of the disease into two groups: (*a*) The "Alimentary form" exists where the sugar is derived from the food, the carbohydrates being misused, the urine containing dextrose only, and giving no reaction with ferric chloride; and finally where the quantity of sugar as shown by the copper test agrees with that shown by the polarimeter, and polarisation fails entirely if the sugar has been removed by copper or fermentation. (*b*) "The Composite form" shows in addition sugar produced by proteid disintegration and other bodies from a similar source, and as a consequence diabetic coma may be brought about. The products of tissue destruction are oxybutyric acid, diacetic acid, and acetone, and these occur in proportion to the severity of the disease. As the first of the three is lævo-rotatory, it will neutralise to some degree the effect of dextrose in an estimation by the polariscope, and the result will be so much less than that given by the copper test. In this way, then, we can estimate both the acid and the sugar present, and the test may be confirmed by removing the dextrose with yeast, and then finding the acid as shown by its lævo-rotatory action alone. The *Lancet* lays stress on the importance in practice of ascertaining which of these types we have to do with. Both prognosis and treatment differ according as one or the other form is present. Pavy[2] at the International Congress insisted that in health there is a very small amount of sugar in the urine and about 1 per 1,000 in the blood. This sugar has a reducing power less than that of dextrose, for indeed the substance is not wholly dextrose. The carbohydrate of the food is normally changed, however, into fat, proteid, and glycogen, and does not enter the circulation as sugar. In the simple form (*a*) of diabetes this change more or less fails to take place. Hence the blood becomes loaded with sugar, and large quantities appear in the urine, while there is not only a loss of food, but various injuries to the tissues take place from its presence in excess. The failure of the intestinal villi and the liver to change the carbohydrates is usually only partial. They can digest, as it were, a smaller amount than in health. If the diet is restricted they may cope with the smaller amount perfectly, and the blood is quickly restored to its condition in health. The assimilative power is then gradually regained in some cases, especially amongst persons advanced in life, and a less restricted diet can be gradually resumed without a return of abnormal sugar to the urine. In the composite form of diabetes the proteid tissue breaks up and sets free sugar and the acetone bodies which appear in the urine. Seegen says that normal blood contains about 0·17 per cent. of glucose, but that an excess leads to sugar in the urine. The cause of this excess is a failure of the transformation of the sugar. He thinks, however, that sugar may appear in the urine without any excess in the blood, and that it is only in the severest forms of diabetes that hyperglycæmia occurs. Here, of course, his view conflicts with that of Pavy altogether.

Williamson[3] points out that his diabetic blood test is invariably correct so far as present experience goes. It can be made when no urine is obtained, or even *post mortem* from the blood of the jugular vein. The reducing substance is contained in urine and in the serum; it is destroyed by the fermenting action of yeast, and is not affected by removing the proteids present. Normal blood in comparatively large quantities has a reducing action, but this is not due to any minute quantity of glucose it may contain, for it is unaffected by fermentation. On the other hand he concludes that the reducing power in diabetic blood, when obtained in the manner he indicates, is due to the excess of glucose it contains. The test should be carried out by adding 20 cubic millimetres of blood to 40 of water. Then a cubic centimetre of methylene blue (1 in 6,000) with 40 cubic millimetres of liquor potassæ are mixed with them, and the whole is boiled for four minutes. The bluish fluid will have changed to a dirty yellow if diabetes is present. The fluids used may be easily measured by the tubes of a hæmocytometer (Gower's), and it is important not to employ an excess of blood if accurate results are to be obtained. Hirschfeld[4] finds that the sugar in the urine during a case of diabetes is a most variable symptom, and tends to spontaneous improvement, though an absolute cure of the disease is rare. If a patient can finally take 200 grammes of carbohydrates daily without sugar appearing, he would regard it as at least a relative cure. He has noticed aggravation of the disease take place after influenza, boils, and colic, but many other intercurrent diseases have often no such result. Cardiac weakness is favourably affected by a more rigid diet. Saundby[5] considers that sugar is formed both from carbohydrates and from proteid food also, so that for every grain of urea excreted two grains of sugar have been used up in the body, derived like the urea from cleavage of proteid. To calculate the amount of sugar thus normally used up, we must add these two sources. Sugar is converted into glycogen by the liver and other tissues, but reconverted and discharged into the blood in small quantities as needed. The capacity of the tissues to consume sugar varies greatly. Diabetes is caused by failure of the storage power of the liver for glycogen and of the consuming power of the tissues for sugar. The former may be due to diseases of the nerves, pancreas, or of the liver itself, or to poisons. The failure of the consuming power of the tissues is secondary, and is probably due to the excessive supply which they have received. It is after the persistent hyperglycæmia has led to this effect on the tissues that diabetes, with failure of nutrition, &c., sets in.

By skilfully arranged diet we must seek to reduce the sugar supply below that which the enfeebled tissues can consume. After some weeks they may regain in favourable cases a part of their activity, and we shall then be able gradually to relax the dietary. Drugs are of inferior importance; yet we often gain some help from opium, bromide, and alkalies. If coma is feared, half an ounce of bicarbonate of soda may be given per rectum in hot water every hour, as well as hypodermics of strychnia. Williamson,[6] in discussing the cause of diabetes, points out that post-mortem examinations may be divided into two groups. In one tumours in the brain, certain diseases of the pancreas, or arteriosclerosis affecting certain parts of the brain, may be found which are the probable causes of the disease. Still these cases are only a minority. In the larger group no pathological conditions are found which are in any way likely to lead to the state. But of late many observations have been recorded tending to show that it is due to an unknown poison introduced from without, possibly through the intestinal canal. Sometimes the disease appears suddenly in healthy children, sometimes it is cured by the occurrence of a fever or other acute disease. The instances of husband and wife both being attacked by it are more numerous than the law of chances will account for, and one well-known poison, phloridzin, will cause a temporary attack. Leo has produced diabetes in animals by the injection of urine from diabetic subjects. Töpfer and Töpper have shown that the injection of the intestinal contents from infected persons into the stomach, intestines, or under the skin of animals produces the disease, and his experiments seem to have been properly guarded by controls. Lepine and Bouleid prepared an extract from the urine in diabetes and pneumonia which has similar effects, while Hammerschlag has isolated a micrococcus for which he claims like powers. If these results are confirmed they will explain what it is that starts the morbid processes, though they may not show what the processes are in themselves. Seegen[7] thinks that in mild cases of diabetes there is merely a failure of the liver to store up glycogen; but in severe ones, where the tissues fail to consume the normal amount, the sugar formed from the albumen and fat, as well as from the carbohydrates, is excreted. Kulz[8] investigated the question whether patients lose all power of assimilating carbohydrates, and found that almost all could cope with some quantity, though this varied greatly with the individual. In his wonderful collection of 700 clinical histories of diabetes, published after his death, one of his editors draws attention to the excessive excretion of ammonia in diabetes, and shows that when this exceeds 2 grammes daily it is of serious prognosis. As to acetone and oxybutyric acid, he believes that the presence of traces is unimportant, but larger amounts are, of course, a grave symptom. Mitra, of Cashmere, gives an analysis of 200 Indian cases.[9] The most common age was from 35 to 45, and the sugar averaged 8 to 10 per cent. Mental overwork, sedentary habits, and an excessive consumption of carbohydrate and saccharine food seem to be the most common antecedents in Indian patients. The poorer classes of Hindoos are not often affected, and the widows, who have the hardest and dullest lives of all, are never attacked. Strict dieting on the European type seems to be impossible, and opium is the drug most employed. Dufourt,[10] in an excellent article on diabetic diet, lays down that in glycosuria the tissues are unable to consume sugar, and therefore they break up fat and albumen in its place. We must calculate therefore the number of calories which the body requires, and give sufficient food to supply this loss. The various classes of foodstuff are not entirely interchangeable, but he does not believe that strict diet ever produces coma, except when its insufficiency causes starvation. Wherever possible, however, he would allow some carbohydrate, but advises an occasional fortnight on strict diet for all.

The nervous symptoms in diabetes are discussed by L. Desbonnets,[11] who draws attention to the transient and permanent hemiplegias and other paralyses, the neuralgias, diplopia, vertigo, headache, and insomnia which are so common. The cerebral state is described as generally apathetic, and the torpor may sometimes deepen into periodic narcolepsy. In late stages we may find hypochondriacal melancholia. The cause of the paralysis is thought to be due to embolism caused by granules of glycogen. Diabetic urine is occasionally found to be of low specific gravity, and even 1002 has been noted. J. B. Herrick[12] thinks these cases are often due to interstitial nephritis coming on intercurrently. Casts will be found in such water. The most important point connected with casts is the appearance of vast numbers of broad granular ones at or before the commencement of coma. When a grey sediment composed of these casts is seen, urgent measures should be taken to prevent the impending coma. A case of pancreatic diabetes due to calculi is reported by E. W. Phillips.[13] The patient, a male, 50, suffered from piles, had lost weight, and became drowsy. There was a history of colic, the motions were offensive, and full of fatty matter, the urine contained sugar but no albumen or acetone and allied substances. Slight peripheral neuritis and lung trouble followed, and the patient gradually sank from exhaustion. D. Le Fevre[14] regards an excess of the night urine over the day secretion as an unfavourable sign, but polyuria with decreased sugar, or high-coloured urine with uric acid, are favourable. Among the eye complications L. W. Alleman[15] refers to paralyses of the extra-ocular muscles, and especially of accommodation. Some writers claim that there is a specific diabetic cataract and retinitis, but that is denied by others. Small punctate hæmorrhages are most suggestive of diabetes, and are often associated with conjunctival ones, and severe iritis is met with. The cutaneous complications, according to S. Sherwell,[16] include dryness of skin, pruritus, eczema, furunculosis, xanthoma diabeticorum, erysipelas, gangrene, dermatitis herpetiformis, and d. blastomyces. Ferrannini[17] finds that lævulose can supply to some extent the place of other carbohydrates in saving the albuminous tissues, and that moderate quantities are not eliminated as sugar. In severe cases, where sugar still appears on a pure meat and fat diet, the use of lævulose will, however, augment the sugar; but if we restrict this diet in quantity, and the sugar disappears, fifty grammes of lævulose may be given on alternate days without causing a return of the sugar. Such doses save the nitrogenous destruction in a much greater degree than equivalent quantities of other carbo-hydrates.

[1] Lancet, July 7. [2] Brit. Med. Jour., August, Ep. [3] Lancet, Aug. 4. [4] Med. Rec., Aug. 25. [5] Practitioner, July. [6] Loc. cit. [7] Brit. Med. Jour., July 14. [8] Loc. cit. [9] Brit. Med. Jour., Sept. 9. [10] Brit. Med. Jour., Sept. 1. [11] Lancet, Nov. 17. [12] Brit. Med. Jour., June 2. [13] Lancet, July 14. [14] Med. Rec., July 21. [15] Loc. cit. [16] Loc. cit. [17] Med. Times and Register, August.

